# The analysis base study on mechanical double enzyme technique for isolating and culturing primary chondrocytes

**DOI:** 10.1016/j.mex.2023.102450

**Published:** 2023-10-18

**Authors:** Wangyuan Yao, Muhammad Fakhar-e-Alam Kulyar, Yanmei Ding, Haitao Du, Yan Zhang, Zhao Zhang, Chuxian Quan, Quan Mo, Jiakui Li

**Affiliations:** College of Veterinary Medicine, Huazhong Agricultural University, Wuhan 430070, PR China

**Keywords:** Chondrocyte, Mechanical-double enzyme, Cartilage, Mechanical Double Enzyme Technique for chondrocytes

## Abstract

The mechanical-double enzyme method was used in the current study to isolate and culture primary chondrocytes from the chicken growth plates. The feasibility and practicability of the approach were determined by using trypan blue staining, toluidine blue staining, PCR, and flow cytometry. The immunofluorescence assay was also used to effectively identify chondrocytes, demonstrating the expression of chondrocyte-specific secreted products (Col-II and Aggrecan). The exterior morphology of chondrocytes was studied at several stages, revealing significant changes in cell shape with each generation. Notably, compared to earlier approaches, the mechanical-double enzyme strategy revealed enhanced cell adhesion and much reduced apoptosis rates. The findings indicate that this novel method has great potential for efficient primary chondrocytes culture, providing important insight into chondrocyte ba research and future applications in cartilage tissue engineering.

The following technical points are included in this method:

• Isolation and culturing primary chondrocytes by a mechanical-double enzyme approach.
• The evaluation of cell adhesion and apoptosis of mechanical double enzyme approach as compared to previous approaches.
• The confirmation of chondrocyte-specific secreted products' expression via toluidine blue staining, PCR, and immunofluorescence assays.

## Graphical abstract

**Figure:**
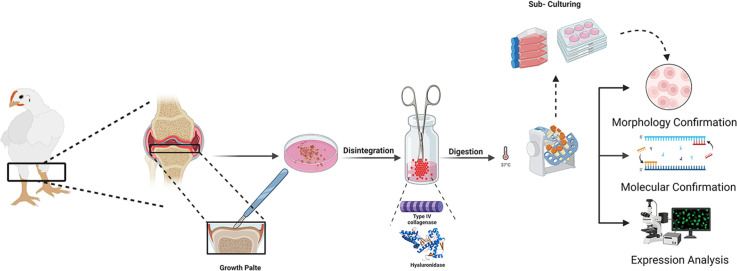

Specifications table

**Table:** 

Subject area:	Veterinary Science and Veterinary Medicine
More specific subject area:	Cell Culture and Tissue Engineering
Name of your method:	Mechanical Double Enzyme Technique for chondrocytes
Name and reference of original method:	Not Applicable
Resource availability:	Not Applicable

## Introduction

Growth plate (GP) chondrocytes lead to subsequent bone formation during the skeletal development [1]. These chondrocytes undergo sequential differentiation to form a resting zone (RZ), proliferative zone (PZ), and hypertrophic zone (HZ) [2], with different cellular morphology and physiology [3]. According to the research, cellular components of the growth plate need to be tightly regulated to achieve normal bone growth for proper differentiation and proliferation [4], [5], [6], [7]. Any disparity in process results in chondrodysplasia or a malformed skeleton, which may lead to different pathologic bone conditions, e.g., tibial dyschondroplasia (TD) [8,9]. Due to the chondrocytes' involvement, these conditions are characterized by non-vascularized, nonviable cartilage in tibial growth plates [10,11]. The difference in chondrocyte's morphology and physiology in various regions has caused significant impediments in research. Furthermore, most of the research and treatments for skeletal diseases are still focused on animal models [12,13]

Regenerative chondrocyte transplantation can effectively repair cartilage damage and cure osteochondrosis [14]. However, the traditional chondrocyte culture techniques are complex and more susceptible to contamination, which may result in chondrocytes' death, low survival rate, and inferior quality [9,15]. Therefore, it is important to develop a more efficient and practical technique for chondrocytes culture *in-vitro* for cartilage repair and cell engineering.

*In-vitro* culture of chondrocytes is an important source to study the physiological and molecular mechanisms. The existing chondrocyte culture models are all based on articular chondrocytes, including primary cultured chondrocytes from chickens, rabbits, rats, and humans [16], [17], [18]. Moreover, these culture models can easily digest with trypsin or collagenase II. So, such damaged growth plate chondrocytes may not be applicable [19]. Therefore, the current study intends to establish an effective technique for isolation and culture of chondrocytes from growth plate *in-vitro* to obtain a large number of chondrocytes with active division and high viability as a theoretical basis for the repair of growth plate and osteochondrosis.

## Method details

### Experimental materials

High-glucose Dulbecco's modified eagle medium (DMEM), Fetal bovine serum, and Trypsin-EDTA solution (0.25%) were purchased from Gibco, USA. Type IV collagenase, Hyaluronidase, and Penicillin-Streptomycin were purchased from Sigma, USA. While CCK-8 kit, and Trypan blue stainwere purchased from Dojindo, Japan and Solar bio, China. Anti-aggrecan Rabbit pAb (ABclonal), Coll1A2 Rabbit pAb (ABclonal), anti-GAPDH Rabbit pAb (ABclonal), HRP goat anti-rabbit IgG (ABclonal), Alexa Fluor® 488 labeled goat anti-rabbit IgG (Servicebio), Cy3 labeled goat anti-rabbit IgG (Servicebio) were purchased for chondrocyte-specific secreted products' expression.

### Cell culture medium

The cell culture medium was prepared from 86% high-glucose DMEM (Dulbecco's modified eagle medium), 12% Australian fetal bovine serum, and 1% antibiotic (Penicillin-Streptomycin). The digestive solution (e.g., 100 mL) was prepared by utlizing 100 mg of type IV collagenase and hyaluronidase to cell culture medium.

### Animals

AA broilers (*n* = 100) were purchased from Xiangyang Zhengda Breeding Company, China. A health assessment was performed on the birds before collecting the tibial bone. Birds suspected of having a disease or physical injuries were excluded. The healthy birds were randomly passed through the euthanasia procedure, having less than 18th days of their age by inhalation drug (carbon dioxide 100%). The operation was carried out in a well-ventilated environment with a closed euthanasia device. The device had good transparency for making observations easy, e.g., checking different reflexes.

### Cell isolation and culture

The chicken's tibia was separated on an aseptic operation table and then put in 75% alcohol for 10 min in order to avoid contamination. The articular cartilage at the top of the tibia was separated with a scalpel until the growth plate was exposed. The growth plate was cut into small pieces and washed twice with PBS solution, then placed in a narrow-necked small vial containing digestive solution (Tip: it is worth mentioning that the growth plates must be separate in this step accurately to avoid other unrelated cells).

The small pieces of growth plate were then cut into a “crumb shape” with sterile ophthalmic scissor. They were, afterward, digested with the digestive media for 11.5 h at 37 °C with 5% CO_2_ in a shaker. (Tip: ophthalmic scissor is recommended at this stage to get convenient and better results). The digestive solution was filtered through a 70 µm cell sieve and centrifuged at 800 rpm for 8 min. The supernatant was then removed and rewashed. Finally, the cells were inoculated into the culture medium at 37 °C with 5% CO_2_ in an incubator. When cells were adherently fused to 80% density, they were digested with 0.25% trypsin-EDTA solution for 40 s at 37 °C with 5% CO_2_. The cells were suspended in a culture medium and re-inoculated onto the other plates.

### Trypan blue staining

A sterile 0.4% trypan blue solution was prepared in advance. Cell suspension and trypan blue solution were mixed at 9:1. The cells and staining solution were gently mixed and stained for 3 min. Afterward, the cells were observed under an inverted microscope and counted with a blood cell counting plate. The survival rate of cells was calculated with the following formula;

$$Living\;cell\;rate\;\left(\%\right)=\frac{\left(total\;number\;of\;cells-number\;of\;blue\;cells\right)}{total\;number\;of\;cells\;\times \;100\%}$$

### Identification of chondrocytes

Chondrocytes were identified by toluidine blue staining, and chondrocyte-specific secretory products (Col-Ⅱ, Aggrecan). The cells were fixed with 4% paraformaldehyde for 15 min. Than 1% toluidine blue was added for 30 min, rinsed with double distilled water to remove the dye. The glacial acetic acid was added for 20 s to clear the nucleus. The glacial acetic acid was washed with PBS. Later, the staining results were observed under an inverted microscope. Besides, the identification of cell-secreted products was observed by PCR assay.

### PCR

The total RNA from growth plate chondrocytes was extracted using Trizol (Life Science) technique and then transcribed to cDNA with a cDNA synthesis kit (Beijing Transgene Biotech). The primary cell cDNA (as a template), 2×Taq Master mix and corresponding primers were added (Table 1). Later, the 5 µl of PCR product was identified by 3% agarose gel electrophoresis.

**Table 1:** Primer used in this study.

Genes	Accession number	Primer sequence (5’-3’)	Product size (bp)
Col-Ⅱ	NM_204426.1	F: ACCTACAGCGTCTTGGAGGA	155
		R: ATATCCACGCCAAACTCCTG	
Aggrecan	NM_ 204955.2	F: TGCAAGGCAAAGTCTTCTACG	248
		R: GGCAGGGTTCAGGTAAACG	
Col-X	NM_00103394.1	F: 5’-ATATCCACGCCAAACTCCTG-3’	471
		R: 5’ TCAGAGGAATAGAGACCATTGGAT-3’	

### Immunoflurescence assay

The cells were rinsed 3 times with PBS and then treated with 4% paraformaldehyde for 20 min at room temperature. Rabbit anti-Col-II and Aggrecan antibodies (1:150) were added and incubated overnight at 4 °C. After thorough washing with PBS, goat anti-rabbit polyclonal antibody labeled with fluorescein isothiocyanate (FITC) was added (1: 100) and then incubated for 1h at room temperature in the dark. DAPI staining labeled nuclei were rinsed with PBS for 30 min. Then after sealing with neutral gum, cells were observed under a fluorescence microscope and photographed.

### Detection of cell apoptosis through Flow cytometry

The cells were trypsinized with EDTA-free trypsin to ensure that the cells were free of toxins. Later on, cells were stained using Annexin V-FITC and Propidium Iodide (PI) solution for 15 min at room temperature according to the manufacturer's instructions, followed by staining using 1 mL of HEPES buffer. Finally, apoptosis was detected at 485 nm using FITC-A fluorescence (525 nm) and PI fluorescence (620 nm).

## Method validation

### Survival of growth plate chondrocytes after mechanical-double enzyme digestion

**Fig. 1:**
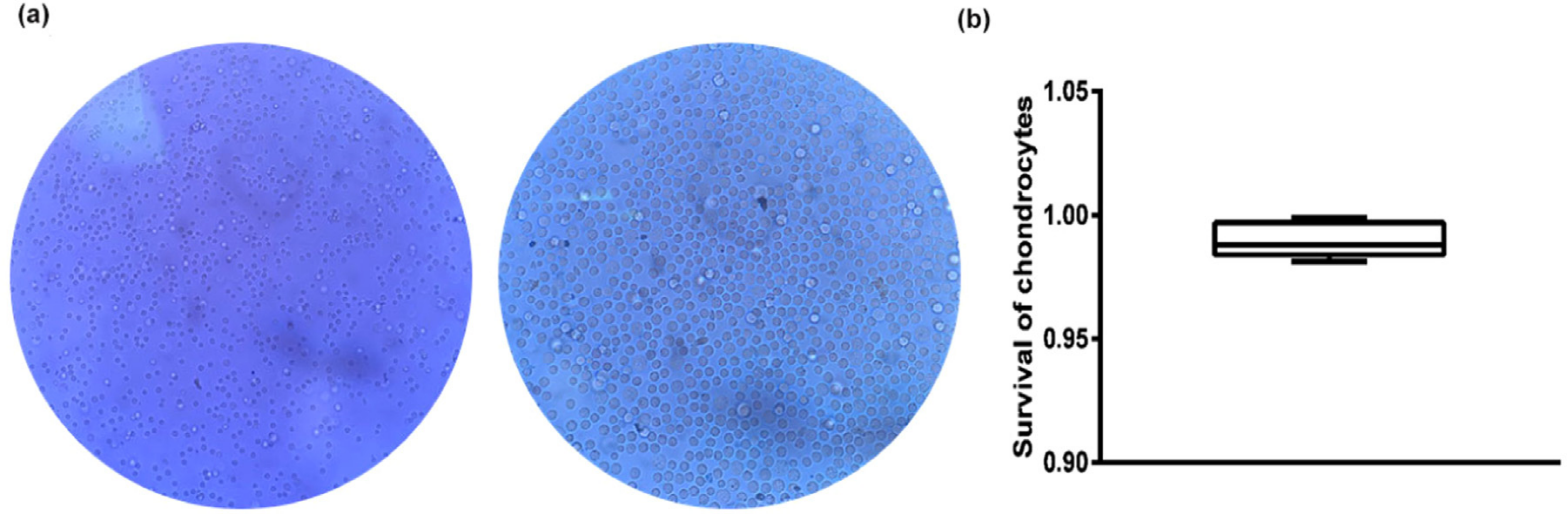
Trypan blue staining for the survival of growth plate chondrocytes. (a) Colorless and transparent cells referred to live cells, while the blue-stained cells referred to dead cells (magnification 40X, 100X). (b) Analysis of cell survival rate.

The mechanical-double enzyme digestion technique's feasibility and practicality were investigated through the survival rate of the chondrocytes by trypan blue staining. The centrifuged and re-suspended overnight digestive solution of chondrocytes with trypan blue staining solution was prepared in advance (Fig. 1a). Chondrocytes were uniformly free in the solution. Living cells were colorless, transparent, and in excellent condition, while the dead cells were dyed blue. The total number of chondrocytes and the number of blue cells were counted separately using a cell counting plate (Fig. 1b). Repeated five times for each group (*n* = 5).

### Identification of chondrocytes

**Fig. 2:**
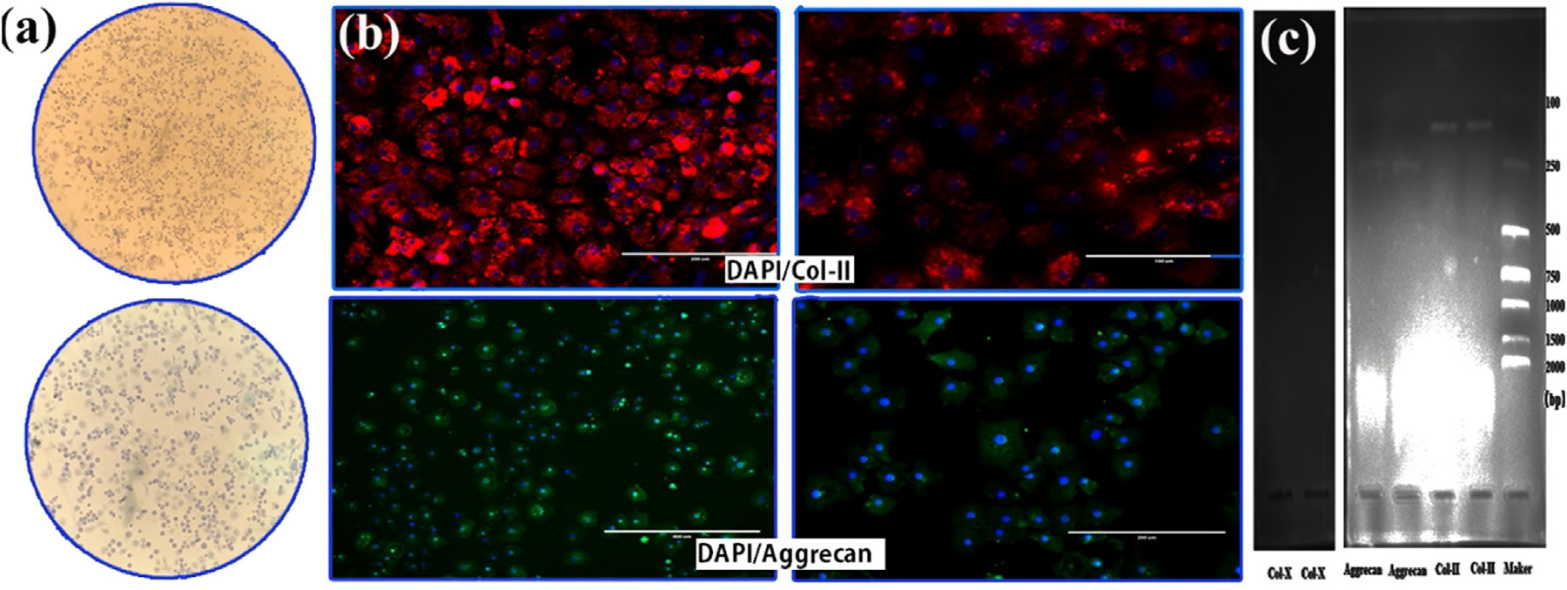
Identification of growth plate chondrocytes. (a) Microscopic observation of toluidine blue staining. The nucleus was stained blue, and there were blue-purple metachromatic particles in the cytoplasm and around the cells (100X, 200X). (b) Microscopic observation of immunofluorescence staining. Col-ll protein was expressed red, Aggrecan was expressed green, and the nucleus of living cells was stained blue (200X, 400X). (c) Detection of chondrocyte-specific secreted products by PCR.

Isolation and culture of chondrocyte cells were determined using toluidine blue staining, immunofluorescence, and PCR of cell-specific secreted products to identify. Toluidine blue is a metachromatic cationic dye to show nuclei and acidic mucopolysaccharides. As shown in Fig. 2a, the nucleus was stained blue. There were blue-purple metachromatic particles in the cytoplasm and around the cells, which were acid mucopolysaccharides secreted by the chondrocytes. Simultaneously, immunofluorescent staining of col-Ⅱ and Aggrecan specific proteins were performed using second-generation growth plate cells. Visible red and green fluorescence throughout the cytoplasm showed that col-Ⅱ and aggrecan proteins were well expressed. The nuclei were oval-shaped and stained blue, proving that the chondrocytes were active (Fig. 2b). Total RNA was extracted from chondrocytes and reverse-transcribed into cDNA. PCR detected the Aggrecan, type II collagen, and type X collagen of chondrocyte secretion products. The 3% agarose gel electrophoresis results showed that the PCR products of proteoglycan and type II collagen were consistent with the expected fragment lengths (248bp and 155bp, respectively). PCR results of type X collagen showed that the tested cells produced a large amount of proteoglycan and type II collagen, but the chondrocytes were not differentiated in the hypertrophic stage (Fig. 2c).

### Morphology of growth plate chondrocytes in different periods

**Fig. 3:**
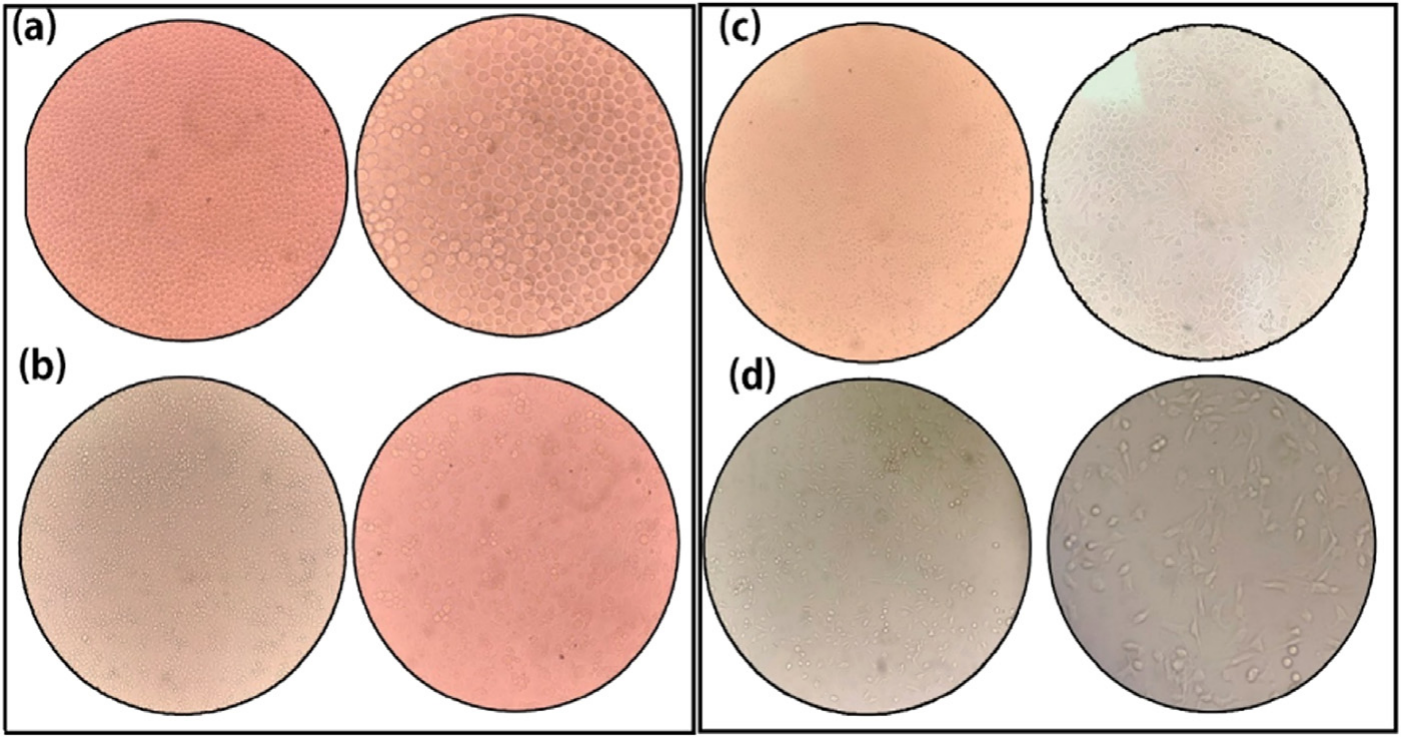
External morphological observation of growth plate chondrocytes in different generations. (a, b) First generation primary chondrocytes; cells were oval, smooth and evenly distributed with evenly distributed like “paving stones”. (c) Second-generation chondrocytes; cells were the same as first-generation, but slightly larger and irregular in appearance. (d) Third-generation chondrocytes; cells gradually became long spindle-shaped with blurred internal structure (100X, 200X).

Morphology of growth plate chondrocytes in different periods was attained for in-depth understanding till the third generation. Chondrocytes isolated by mechanical- double enzyme technique were seeded into culture plates. The primary chondrocytes' morphology was oval; the surface was smooth and evenly distributed (Fig. 3a). When primary cells adhered to 80% of the culture plate, a 0.25% Trypsin-EDTA solution was used to separate and re-cultured in culture plates for obtaining second generation cells. The chondrocytes adhered to the surface, gradually changed, showing a little different shape (Fig. 3b). The morphology of second-generation chondrocytes was slightly larger (Fig. 3c), suggesting the use of chondrocytes as model cells at this stage for scientific experiments. Unusually, the third-generation chondrocytes' external morphology gradually became long spindle-shaped (Fig. 3d), and the internal structure was subsequently lysed until apoptosis.

### Comparison of the mechanical double enzyme with previous methods

**Fig. 4:**
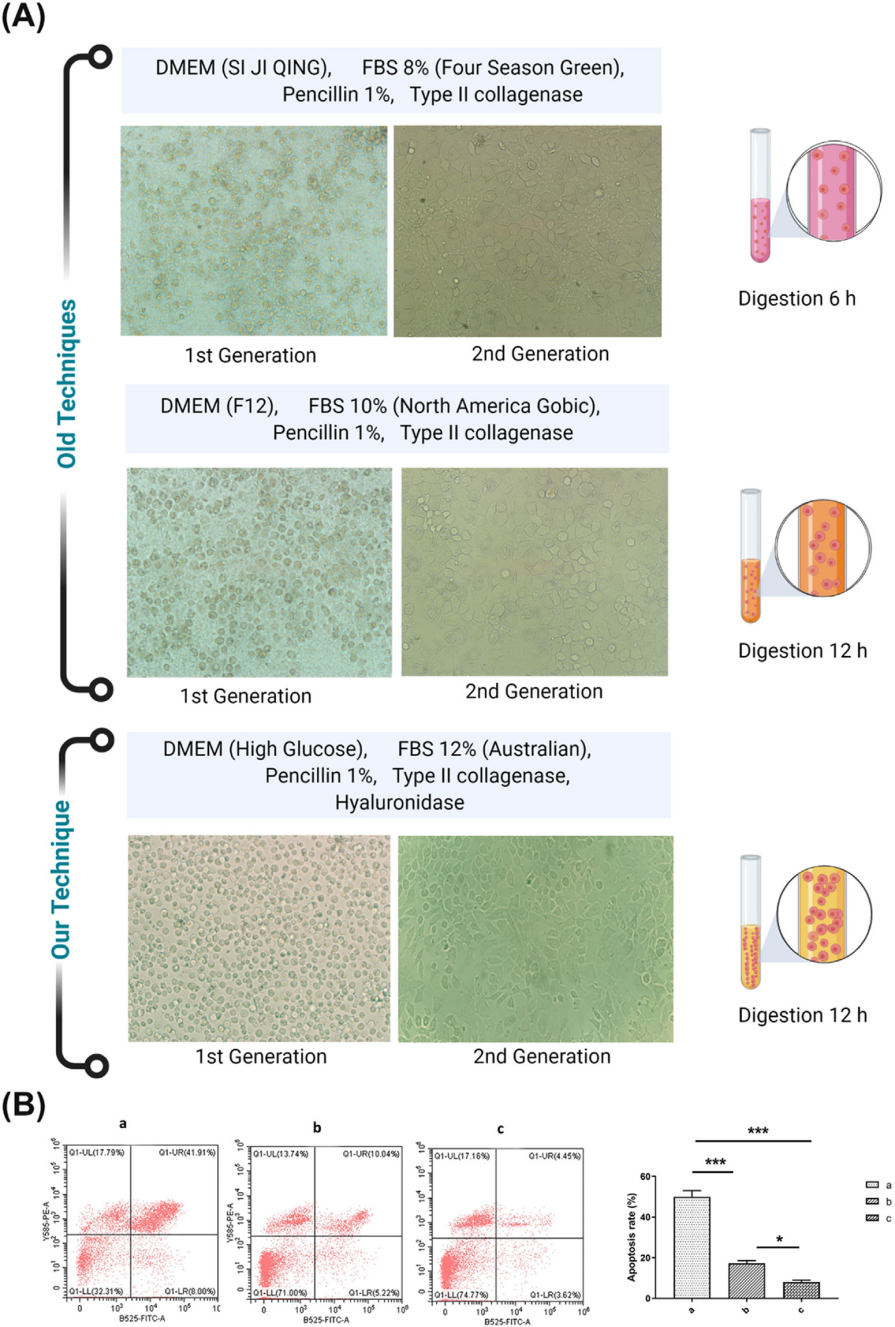
Comparison of mechanical double enzyme method with previously developed methods for chondrocytes culture. (A) Morphological comparison of chondrocytes under a light microscope. (B) Analysis of apoptotic cell percentage among different techniques through flow cytometry. a; DMEM (SI JI QING), FBS 8% (Four Season Green), Pencillin 1%, Type II collagenase. b; DMEM (F12), FBS 10% (North America Gobic), Pencillin 1%, Type ll collagenase. c; DMEM (High Glucose), FBS 12% (Australian), Pencillin 1%, Type II collagenase, Hyaluronidase.

Cells were checked microscopically daily to monitor growth rates and confluency. More adherent cells were attached to the bottom of the flask and showed an exact adherent morphology in our method compared to previous techniques. Chondrocytes were principally irregular and dwindled in old techniques (Fig. 4A). The passage number was also gone through. It was recorded as high without undergoing genetic drift in current technique. Moreover, the apoptotic rate was confirmed between old and our developed techniques. According to findings, our developed technique has less apoptosis percentage of cells after 48 h of incubation time (Fig. 4B). All these results revealed a positive bias of our method. Indeed, the best results were obtained by culturing chondrocytes through a mechanical double enzyme *in-vitro*.

## Ethics statements

All experimental animal maintenance, handling, and procedures were performed following Huazhong Agricultural University's ethics committee (Approval No. 31273519) and the National Institutes of Health guide for the care and use of laboratory animals (NIH Publications No. 8023, revised 1978).

## CRediT authorship contribution statement

**Wangyuan Yao:** Conceptualization, Methodology, Writing – original draft. **Muhammad Fakhar-e-Alam Kulyar:** Software, Investigation, Methodology, Conceptualization, Validation, Writing – review & editing. **Yanmei Ding:** Validation, Data curation. **Haitao Du:** Visualization, Investigation. **Yan Zhang:** Validation, Formal analysis. **Zhao Zhang:** Visualization, Formal analysis. **Chuxian Quan:** Formal analysis, Investigation. **Quan Mo:** Formal analysis, Resources, Visualization. **Jiakui Li:** Supervision, Resources, Funding acquisition, Project administration.

## Declaration of Competing Interest

The authors declare that they have no known competing financial interests or personal relationships that could have appeared to influence the work reported in this paper.

## Data Availability

Data will be made available on request.
